# Effect of Extruding Full-Fat Soy Flakes on Trans Fat Content

**DOI:** 10.1155/2014/427423

**Published:** 2014-08-19

**Authors:** Hongxia Feng, Xiaonan Sui, Yunhe Chang, Baokun Qi, Yan Zhang, Yang Li, Lianzhou Jiang

**Affiliations:** ^1^College of Food Science, Northeast Agricultural University, Harbin 150030, China; ^2^School of Science, The National University of Singapore, Singapore 119077; ^3^Research Institute of Food and Beverage, Wahaha Group Co. Ltd., Hangzhou 310018, China

## Abstract

To evaluate the effects of extrusion process on the trans fatty acids (TFAs) formation in soybean crude oils, three different extrusion parameters, namely, extrusion temperature (80–160°C), feed moisture (10–26%), and screw speed (100–500 rpm), were carried out. It was found that only five different types of TFAs were detected out using gas chromatography-mass spectrometry. Before the extrusion started, the initial amount of total TFAs was 3.04 g/100 g. However, after extruding under every level of any variable, the total amounts of TFAs were significantly higher than those in the control sample (*P* < 0.05). For example, taking the effect of extrusion temperature into account, we can find that the highest amount of total of trans fatty acid (TTFA) was 1.62 times the amount of that in the control sample, whereas the lowest amount of TTFA was 1.54 times the amount of that in the control sample. Importantly, it was observed that the amounts of every type of trans fatty acid were not continuously increasing with the increase of the level of any extrusion variable. This phenomenon demonstrated that the formation and diversification were intricate during extruding process and need to be further studied.

## 1. Introduction

Trans fatty acids (TFAs) are geometrical isomers of unsaturated fatty acids containing nonconjugated carbon-carbon double bonds possessing the* trans*-configuration [1]. Epidemiologic and clinical studies have revealed that the consumption of TFAs can lead to a higher risk of coronary heart disease, sudden death, and diabetes mellitus [2, 3]. Therefore, a minimal intake of TFAs was recommended to lower the associated health risks [2–4].

Previous studies have suggested that low level of TFAs in diet might derive from microbial biohydrogenation in the digestive tract of ruminant animals, whilst high level of TFAs is mainly produced through the consumption of partially hydrogenated edible oils in processed products [5, 6]. TFAs can also be produced during the oil refining process owing to the high temperature used in the deodorization procedure [7]. Crude oils are commonly used as raw materials for hydrogenation and refinement to prepare edible oils, and the consequent TFA content of the edible oils is affected by the original TFA level in the crude oil feedstock. It is necessary to control the TFA content of crude oil feedstock. However, to increase oil yields, oil seeds are often subjected to pretreatments, such as flaking, cooking, extruding, and ultrasound, prior to the extraction process. A recent work showed that the extrusion of oil seeds may lead to a complete disruption of cellular structure in favor of increasing the oil extraction yield [8]. Extrusion pretreatment process is still a complicated multi-input-output system and has a complicated impact on the product properties. Several studies have reported the influence of extrusion on the yield and the physicochemical properties of crude oils [9–14]. Lamsal et al. reported that the oil extraction recovery was increased from 68% to 88% after treatment with extrusion [12]. Wang and Johnson found that crude soybean oil from extruding process was low in phosphorus, FFA contents, and the oxidative stabilities [14]. In addition, the nutritional value of lipids could be affected during extrusion as a result of oxidation, hydrogenation, isomerization, or polymerization. According to Maga, the extent of hydrogenation and* cis*- to* trans*-isomerization of fatty acids that takes place during extrusion is too small to be nutritionally significant [15, 16]. Furthermore, Xu et al. observed that a high-temperature-microtime-treated diet with extruded soybean increased the level of* trans*-10,* cis*-12 conjugated linoleic acid, which may inhibit lipogenesis in the intramuscular fat of Holstein steers [17]. However, the effect of extrusion variables on TFA content in crude oil has rarely been studied. Therefore, the primary purpose of the study was to determine the TFAs content in the crude oil.

Nowadays crude oils are conventionally extracted using an organic solvent such as hexane or ether which might have environmental and safety implications. With concerns on safety and environmental issues, numerous research groups have been interested in using the enzyme-assisted aqueous extraction processing (EAEP) method, as an environmentally friendly alternative to solvent extraction. For EAEP, water is used as an extracting medium to remove oil as an emulsion or free oil unlike organic solvents, which dissolve the oil. Simultaneously, the enzymatic hydrolysis of proteins in the cell walls and pseudomembranes surrounding the oil bodies is conducive to enhance the oil yield [8]. Moreover, the crude oil obtained by the method of EAEP was reported to be partially degummed oil with very low phosphorus content and, thus, was suitable for physical refining [18]. Due to these advantages, in the present study, EAEP was applied for the extraction of the crude oil. In order to enhance the oil yield, a twin-screw extruder was applied prior to the EAEP of crude oil from full-fat dehulled soybean flakes. The aim of this work was to study the effect of extrusion technology on the formation and the content of TFAs in the crude oil.

## 2. Materials and Methods

### 2.1. Materials and Reagents

Full-fat, dehulled soybean flakes containing 7.3% moisture and 16.5% oil were obtained from Fengzheng Soybean Food Co. Ltd. (Dunhua, China) in 2013. Protex 6L (2.4 AU/g, from* Bacillus licheniformis*) was purchased from Novo-Nordisk A/S (Bagsvaerd, Denmark).* cis-/trans*-Linoleic acid methyl ester mix (Catalog number 47791), linolenic acid methyl ester isomer mix (Catalog number 47792),* trans*-6-octadecenoic methyl ester (Catalog number 47199), methyl elaidate (Catalog number 45119-1mL), and* trans*-11-octadecenoic methyl ester (Catalog number 46905-U) were purchased from Sigma-Aldrich (St. Louis, USA). All other chemicals and reagents were obtained from commercial suppliers and are of analytical grade.

### 2.2. Sample Preparation

Dehulled soybean flakes were extruded using an EV-25 twin-screw extruder (Clextral, made in France) in the set conditions described in Table 1. In order to study the effect of the three variables, namely, extrusion temperature, feed moisture, and screw rotational speed, extruding was implemented via three groups of run. When one variable was controlled, the other two were fixed at a certain value. For example, to study the effect of extrusion temperature on the content of trans fatty acid in the oil from the sample, the two variables of feed moisture and screw rotational speed were fixed at the value of 14% and 200 rpm, respectively, according to the parameters factory usually applied. Then the extrusion temperature (ranged from 80 to 160°C) was adjusted to the set value one by one and from low to high. During the adjustment, the extruder was continuously operational. Until the extruder was stabilized at the set condition (e.g., 80°C), the extrudates were sampling. After the extrusion pretreatment process, the press strips were allowed to cool to ambient temperature and ground into a fine powder. The powder was subsequently used for oil extraction using the EAEP method according to the previous work of our group [19]. The extracting experiment for preparing oil sample from extrudates obtained from each extruding run was conducted in triplicate. The oil samples obtained were stored in dark bottles without headspace at –20°C prior to analysis.

### 2.3. Determination of Trans Fatty Acid Content in Soybean Oil

Fatty acid methyl esters (FAMEs) were prepared according to the method previously reported by our group [19]. After* O*-methylation, the FAMEs were separated and quantified by gas chromatography-mass spectrometry (GC-MS) using an Agilent model 6890-5973 GC-MS equipped with a flame ionization detector and HP-88 fused silica capillary column (100 m × 0.25 mm i.d. × 0.2 *μ*m film thickness). Helium was used as the carrier gas at a flow rate of 1 mL/min and the detector temperature was 250°C. The split ratio was 30 : 1 and the column temperature was programmed as follows: (i) the initial oven temperature was 80°C and it was maintained at 80°C for 5 min; (ii) the oven temperature increased to 150°C at a rate of 10°C/min and was maintained at 150°C for 2 min; (iii) the oven temperature increased to 230°C at a rate of 5°C/min and was maintained at 230°C for 10 min. The individual fatty acids were identified by comparison of the retention times with preprepared standards and quantified using an external standard. The analysis of each oil sample was conducted in triplicate, and the mean value was used for the later variance analysis.

### 2.4. Statistical Analysis

Experiments were performed in triplicate and results were expressed as mean ± standard deviation (SD). Data were subjected to the one-way analysis of variance (ANOVA) and the significance was defined at *P* < 0.05. The variance analysis was conducted using SPSS (statistical program for social sciences, SPSS Corporation, Chicago, IL, USA; version 16.0 for Windows).

## 3. Results and Discussion

### 3.1. GC-MS Analysis of the TFA Standard Mixtures

**Figure 1:**
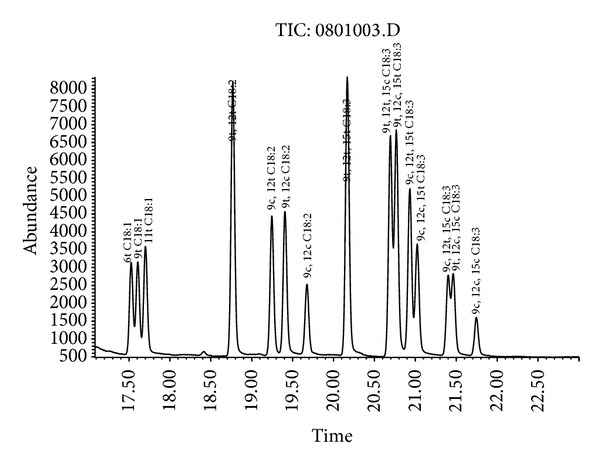
The GC-MS chromatogram of TFA methyl ester standards. Retention time (RT; min) of each standard compound was as follows: C18:1, 6t (17.32); C18:1, 9t (17.41); C18:1, 11t (17.50); C18:2, 9t, 12t (18.54); C18:2, 9c, 12t (19.02); C18:2, 9t, 12c (19.17); C18:2, 9c, 12c (19.45); C18:3, 9t, 12t, 15t (19.92); C18:3, 9t, 12t, 5c (20.45); C18:3, 9t, 12c, 15t (20.53); C18:3, 9c, 12t, 15t (20.68); C18:3, 9c, 12c, 15t (20.78); C18:3, 9c, 12t, 15c (21.15); C18:3, 9t, 12c, 15c (21.22); C18:3, 9c, 12c, 15c (21.50).

The GC-MS chromatogram of the TFA methyl ester standards is shown in Figure 1. A total of 16 peaks, including 2 ×* cis-*unsaturated fatty acids, 13 × trans fatty acids, and internal standards (C17:0; RT = 19.78), were resolved within 22 min. We failed to achieve baseline separation of the* trans*-isomers of oleic acid (peaks: 6t, 9t, and 11t) and linolenic acid (peaks: 9t, 12t, and 15c; 9t, 12c, and 15t; 9c, 12t, and 15t + 9c, 12c, and 15t; and 9c, 12t, and 15c) through a single run of chromatography. The TFAs are eluted prior to their corresponding* cis*-isomers, because a capillary column was used as a cyanoalkyl polysiloxane polar stationary phase. For example, the* trans-*isomers of linoleic acid (C18:2, 9t, 12t; C18:2, 9c, 12t) were eluted prior to the* cis*-isomers of linoleic acid (C18:2, 9c, 12c). The partially overlapped peaks corresponding to* trans*-isomers did not affect the quantitative analysis, since for food labeling these isomers are regarded as a representation of the total amount of* trans*-oleic acid or* trans*-linolenic acid. Nevertheless, this method is an improvement in terms of retention time compared to those previously reported [20].

#### 3.1.1. Effect of Different Extruding Parameters on the TFA Content

In this study, we focused on the formation of the TFAs bearing 18 carbon atoms which are the most representative in the samples. The main TFA isomers found in the control samples were isomers of C18:1, 9t (0.57 g/100 g); C18:2, 9c, 12t (0.64 g/100 g); C18:2, 9t, 12c (0.11 g/100 g); C18:3, 9t, 12t, 15c/9t, 12c, 15t (0.68 g/100 g); and C18:3, 9c, 12c, 15t (1.05 g/100 g) (Tables 2–4). The TFAs of C18:1, 6t; C18:1, 11t; C18:2, 9t, 12t; C18:3, 9t, 12t, 15t; C18:3, 9c, 12t, 15t; C18:3, 9c, 12t, 15c; and C18:3, 9t, 12c, 15c were not detected in all the tested samples. In our study, the initial amount of total TFAs in the control samples was 3.04 g/100 g of extracted soy oil (Tables 2–4). In a previous study, the amounts of TFAs were reported to be 0.26–0.82 g/100 g in soybean oil [21]. Higher TFA content (1.15–3.11 g/100 g) in soybean oil was also reported [4]. Hou et al. also reported the values of TFAs in rapeseed oil (1.37–3.82 g/100 g), sunflower oil (1.41–2.10 g/100 g), and corn oil different range (2.01–4.76 g/100 g). By comparing the various* trans*-forms of fatty acids, oleic acid was the most susceptible to formation during extruding process.

#### 3.1.2. Effect of Extrusion Temperature

The changes in the TFA composition of soybean oil extracted from extrudates under various extrusion temperatures (80, 100, 120, 140, and 160°C) were shown in Table 2. Compared to the control samples, no continuous increase or decrease in TFA content was observed with increasing extrusion temperature. This was due to the instability of different types of TFAs [22] and discontinuity of chamber pressure. After extrusion at 80°C, the sharp increase of total* trans*-linoleic acid (by 1.70 g/100 g) could mostly be attributed to the increase of chamber pressure from 1.0133 bars to 9.9113 bars. However, the amounts of* trans-*oleic acid (C18:1, 9t) after extruding at 100°C were significantly increased by 1.68 g/100 g, whereas the total amounts of* trans*-linoleic acid were significantly decreased by 1.63 g/100 g. This shift phenomenon seemly suggested that part of* trans*-linoleic acid was transformed into* trans*-oleic acid. Although diverse changes occurred for different types of TFAs, the total amounts of TFA reached a maximum of 4.91 g/100 g after extrusion at 160°C. It could be found that, among these different levels of the extrusion temperature, the maximum amount of TTFA was 1.62 times that in the control sample, while the minimum amount of TTFA was 1.54 times that in the control sample. Although the change of every type of trans fatty acid was fluctuant, the range of this variation was not very great during extrusion with increasing temperature. Since the* cis-* to* trans*-isomerization may occur at 150°C [23], the formation of trans fatty acids was likely exacerbated by the combined effects of pressure and high temperature. A previous study reported that only 1% to 2% of the unsaturated fatty acids were converted to the* trans*-form during the extrusion of cornmeal [16, 24]. After increasing extrusion temperature from 155°C to 171°C, the amount of trans fatty acid increased from 1% to 1.5% [16, 25].

#### 3.1.3. Effect of Feed Moisture

The changes in TFA composition of soybean oil extracted from extrudates under various feed moisture levels (10, 14, 18, 22, and 26%) were presented in Table 3. After extrusion at a low content of feed moisture (10%), a distinct increase of 1.27 g/100 g and 0.18 g/100 g was observed for* tran*s-oleic acid (C18:1, 9t) and* trans*-linolenic acid (C18:3, 9c, 12c, 15t), respectively. This is a result of a higher shear force, chamber pressure, and longer residence time at a lower moisture level [26]. After increasing the feed moisture level to 14%, the amounts of all the five types of TFAs exhibited an increasing trend. However, when the feed moisture was increased continuously, the total amount of these TFAs began to reduce, probably because the force, required to push the wet mass through the die, was reduced when increasing the feed moisture content. Simultaneously, the friction between raw material and screw shaft started to decrease [27]. Similarly, the minimum and maximum of the TTFA with different feed moisture were compared with those in the control sample. This variance based on the TTFA in the control sample, and it ranged from 1.36 to 1.60 times that in the control sample. The effect of the feed moisture was shown to be bidirectional, which was confirmed by the result of the two opposite effects of moisture content on the rheology of the feed material in the barrel and die of the extruder [28]. Surprisingly, undetected phenomenon of the* trans*-linoleic acid (C18:2, 9t, 12c) appeared twice, when the extrusion feed moisture was 10% and 22%, respectively. These results indicated that the* trans*-linoleic acid (C18:2, 9t, 12c) was not stable, which was confirmed by a previous study [29]. The calculated energy difference between the* cis-* and* trans*-linoleic acids was equal to 13.2 kJ/mol, whereas the energy difference between the* cis-* and* trans*-forms of oleic acid was 37 kJ/mol.

#### 3.1.4. Effect of Screw Rotational Speed

The changes in TFA composition of soybean oil extracted from extrudates under various screw rotational speeds (100, 200, 300, 400, and 500 rpm) were shown in Table 4. Again, the levels of the main five types of TFAs in soybean oil changed significantly after altering the screw rotational speeds. A sharp increase of 1.16 g/100 g occurred for* trans*-oleic acid (C18:1, 9t) when extruding at a screw rotational speed of 100 rpm. Although the screw rotational speed was low, the high chamber pressure might promote the isomerization of the different TFAs. Moreover, when increasing the screw rotational speed to 200 rpm, the levels of* trans*-oleic acid (C18:1, 9t) and the total TFAs were up to their maximum of 2.09 and 4.87 g/100 g, respectively. This phenomenon was the same as that observed for the effect of feed moisture content. However, the maximum levels of the total linoleic acid (0.84 g/100 g) and the total* trans*-linolenic acid (2.00 g/100 g) were observed after extruding at a screw rotational speed of 300 rpm. The maximum level for any type of TFA was not received at the maximum of screw rotational speed. This result proved that TFAs could undergo degradation under the severe experimental conditions used [20]. Meanwhile, compared with the effect of feed moisture, the same times of the amount of TTFA in the control were found, respectively, for the minimum (1.36) and maximum (1.60) of TTFA under different screw rotational speeds.

## 4. Conclusion

Dehulled full-fat soybean flakes were extruded for the extraction of soybean oil to study the effect of the extrusion process parameters, barrel temperature, feed moisture, and screw rotational speed, on the content of trans fatty acid (TFA). By comparing the results shown above, we can conclude that extrusion technology could increase the formation of TFA. All the levels of the extrusion variables caused diverse fluctuation of the amount of TFA. Although the gap between the minimum and the maximum of total amount of TFA was smaller under the variable of extrusion temperature than that of the other two variables (feed moisture and screw rotational speed), the increased magnitude with effect of the first level of extrusion temperature was greater than that of the other two variables. In other words, the sudden extrusion temperature could transiently increase the formation of TFA in the oil. In summary, the content of TFA in soybean oil could be controlled by changing the extrusion parameters. To comprehensively explore the effect of the extrusion process, further studies are needed to investigate the thermal properties of the TFAs and explore appropriate extrusion condition for the lowest amount of TFA.

## Figures and Tables

**Table 1:** The extrusion parameters for full-fat, dehulled soybean flakes.

Groups of run	Variables^a^		
	*T*	*F*	*R*
1	80	14	200
	100	14	200
	120	14	200
	140	14	200
	160	14	200

2	100	10	200
	100	14	200
	100	18	200
	100	22	200
	100	26	200

3	100	14	100
	100	14	200
	100	14	300
	100	14	400
	100	14	500

^a^Variables are as follows: *T*: extrusion temperature (°C); *F*: feed moisture (% wet basis); *R*: screw rotational speed (rpm).

**Table 2:** Trans fatty acid composition in soybean oil extracted by EAEP from extrudates at various extrusion temperatures under the condition of fixed feed moisture (14%) and rotational screw speed (200 rpm).

Fatty acids^B^ (g/100 g)	Extrusion temperature (°C) (pressure (bar))					
	Control^A^ (1.0133)	80 (9.9113)	100 (2.2745)	120 (2.1050)	140 (8.0125)	160 (12.0500)
C18:1, 9t	0.57 ± 0.01^d^	0.41 ± 0.01^e^	2.09 ± 0.04^a^	1.78 ± 0.02^c^	1.91 ± 0.03^b^	2.07 ± 0.04^a^
C18:2, 9c, 12t	0.64 ± 0.01^d^	1.61 ± 0.02^a^	0.74 ± 0.01^c^	0.82 ± 0.01^b^	0.84 ± 0.01^b^	0.76 ± 0.00^c^
C18:2, 9t, 12c	0.11 ± 0.01^c^	0.84 ± 0.01^a^	0.07 ± 0.01^d^	ND^C^	0.21 ± 0.01^b^	0.09 ± 0.00^d^
Total *trans*-C18:2	0.74 ± 0.01^d^	2.44 ± 0.05^a^	0.81 ± 0.01^c^	0.82 ± 0.01^c^	1.05 ± 0.01^b^	0.85 ± 0.01^c^
C18:3 (9t, 2t, 15c + 9t, 12c, 15t)	0.68 ± 0.01^b,c^	0.69 ± 0.01^b^	0.68 ± 0.01^b,c^	0.70 ± 0.01^a^	0.69 ± 0.01^b^	0.67 ± 0.01^c^
C18:3, 9c, 12c, 15t	1.05 ± 0.01^e^	1.14 ± 0.01^d^	1.30 ± 0.02^b^	1.40 ± 0.02^a^	1.25 ± 0.02^c^	1.32 ± 0.02^b^
Total *trans*-C18:3	1.73 ± 0.02^d^	1.82 ± 0.03^c^	1.98 ± 0.04^b^	2.10 ± 0.04^a^	1.94 ± 0.04^b^	1.99 ± 0.04^b^
TTFA^D^	3.05 ± 0.06^d^	4.67 ± 0.09^b,c^	4.87 ± 0.10^a,b^	4.71 ± 0.09^b,c^	4.90 ± 0.10^a^	4.91 ± 0.10^a^

^A^Control: fresh oil from unextruded soybean flakes.

^B^Mean of duplicate analyses ± standard deviation.

^C^ND: not detected.

^D^TTFA: total of trans fatty acids.

^a–f^Symbols bearing different letters in the same row are significantly different (*P* < 0.05).

**Table 3:** Trans fatty acid composition of soybean oil extracted by EAEP from extrudates at various feed moisture levels under the condition of fixed extrusion temperature (100°C) and rotational screw speed (200 rpm).

Fatty acids^B^ (g/100 g)	Feed moisture (%) (pressure (bar))					
	Control^A^ (1.0133)	10 (16.0825)	14 (2.2745)	18 (2.8938)	22 (0.2213)	26 (0.2413)
C18:1, 9t	0.57 ± 0.01^e^	1.84 ± 0.03^c^	2.09 ± 0.04^a^	2.00 ± 0.04^a,b^	1.71 ± 0.03^d^	1.96 ± 0.04^b^
C18:2, 9c, 12t	0.64 ± 0.01^e^	0.72 ± 0.01^b^	0.74 ± 0.01^b^	0.69 ± 0.01^c^	0.63 ± 0.01^e^	0.78 ± 0.01^a^
C18:2, 9t, 12c	0.11 ± 0.01^b^	ND^C^	0.07 ± 0.01^c^	0.07 ± 0.01^c^	ND	0.21 ± 0.01^a^
Total *trans*-C18:2	0.74 ± 0.01^c^	0.72 ± 0.01^c^	0.81 ± 0.02^b^	0.76 ± 0.01^c^	0.63 ± 0.01^d^	0.99 ± 0.02^a^
C18:3 (9t, 12t, 15c + 9t, 12c, 15t)	0.68 ± 0.01^a^	0.68 ± 0.01^a^	0.68 ± 0.01^a^	0.67 ± 0.01^a^	0.66 ± 0.01^a^	0.67 ± 0.01^a^
C18:3, 9c, 12c, 15t	1.05 ± 0.02^d^	1.23 ± 0.02^b^	1.30 ± 0.02^a^	1.10 ± 0.02^c^	1.14 ± 0.02^c^	1.10 ± 0.02^c^
Total *trans*-C18:3	1.73 ± 0.03^c^	1.91 ± 0.00^a,b^	1.98 ± 0.09^a^	1.77 ± 0.05^b,c^	1.80 ± 0.08^b^	1.78 ± 0.06^b,c^
TTFA^D^	3.04 ± 0.06^e^	4.47 ± 0.08^c^	4.87 ± 0.10^a^	4.53 ± 0.08^b,c^	4.14 ± 0.07^d^	4.73 ± 0.09^a,b^

^A^Control: fresh oil from unextruded soybean flakes.

^B^Means of duplicate analyses ± standard deviation.

^C^ND: not detected.

^D^TTFA: total of trans fatty acids.

^a–f^Symbols bearing different letters in the same row are significantly different (*P* < 0.05).

**Table 4:** Trans fatty acid composition of soybean oil extracted by EAEP from extrudates at various screw rotational speeds under the condition of fixed extrusion temperature (100°C) and feed moisture (14%).

Fatty acids^B^ (g/100 g)	Screw rotational speed (rpm) (pressure (bar))					
	Control^A^ (1.0133)	100 (14.9382)	200 (2.2745)	300 (0.2432)	400 (0.2841)	500 (0.4299)
C18:1, 9t	0.57 ± 0.01^e^	1.73 ± 0.03^c^	2.09 ± 0.04^a^	1.62 ± 0.03^d^	1.73 ± 0.03^c^	1.99 ± 0.04^a,b^
C18:2, 9c, 12t	0.64 ± 0.01^c^	0.71 ± 0.01^b^	0.74 ± 0.01^a^	0.75 ± 0.01^a^	0.61 ± 0.01^c^	0.75 ± 0.01^a^
C18:2, 9t, 12c	1.05 ± 0.02^a^	0.08 ± 0.01^b^	0.07 ± 0.01^b^	0.08 ± 0.01^b^	0.10 ± 0.01^b^	0.08 ± 0.01^b^
Total *trans*- C18:2	0.74 ± 0.01^c^	0.78 ± 0.01^b^	0.81 ± 0.02^a,b^	0.84 ± 0.02^a^	0.70 ± 0.00^d^	0.82 ± 0.00^a^
C18:3 (9t, 12t, 15c + 9t, 12c, 15t)	0.68 ± 0.01^a^	0.66 ± 0.01^a^	0.68 ± 0.01^a^	0.69 ± 0.01^a^	0.65 ± 0.01^a^	0.68 ± 0.01^a^
C18:3, 9c, 12c, 15t	1.05 ± 0.02^c^	1.20 ± 0.02^b^	1.30 ± 0.02^a^	1.32 ± 0.02^a^	1.05 ± 0.02^c^	1.24 ± 0.02^b^
Total *trans*-C18:3	1.73 ± 0.03^c^	1.87 ± 0.03^a,b^	1.98 ± 0.04^a^	2.00 ± 0.04^a^	1.70 ± 0.03^c^	1.93 ± 0.05^a^
TTFA^C^	3.04 ± 0.06^e^	4.38 ± 0.08^c^	4.87 ± 0.10^a^	4.46 ± 0.08^c^	4.13 ± 0.07^d^	4.74 ± 0.09^b^

^A^Control: fresh oil from unextruded soybean flakes.

^B^Means of duplicate analyses ± standard deviation.

^C^TTFA: total of trans fatty acids.

^a–f^Symbols bearing different letters in the same row are significantly different (*P* < 0.05).
